# Tailoring optical properties and stimulated emission in nanostructured polythiophene

**DOI:** 10.1038/s41598-019-43719-0

**Published:** 2019-05-14

**Authors:** Alberto Portone, Lucia Ganzer, Federico Branchi, Rodrigo Ramos, Marília J. Caldas, Dario Pisignano, Elisa Molinari, Giulio Cerullo, Luana Persano, Deborah Prezzi, Tersilla Virgili

**Affiliations:** 1 Dipartimento di Matematica e Fisica “Ennio De Giorgi”, Università del Salento, Via Arnesano I-73100, Lecce, Italy; 2 NEST, Istituto Nanoscienze-CNR and Scuola Normale Superiore, Piazza S. Silvestro 12, I-56127 Pisa, Italy; 3 Dipartimento di Fisica Politecnico di Milano, I-20132 Milano, Italy; 4 Instituto de Física, Universidade de São Paulo, 05508-900 São Paulo, SP Brazil; 5 Dipartimento di Fisica, Università di Pisa, Largo B. Pontecorvo 3, I-56127 Pisa, Italy; 6 Istituto Nanoscienze CNR-NANO-S3, Via Campi 213/A, I-41125 Modena, Italy; 7 Dipartimento di Scienze Fisiche, Informatiche e Matematiche, Università di Modena e Reggio Emilia, Via Campi, 213/a, I-41125 Modena, Italy; 8 IFN-CNR, c\o Dipartimento di Fisica, di Milano, I-20132 Milano, Italy; 9 Present Address: Centro Universitario das Faculdades Metropolitanas Unidas, São Paulo, SP Brazil; 10 Present Address: Max Born Institute, Max-Born-str. 2A, 12489 Berlin, Germany

**Keywords:** Materials science, Optics and photonics

## Abstract

Polythiophenes are the most widely utilized semiconducting polymers in organic electronics, but they are scarcely exploited in photonics due to their high photo-induced absorption caused by interchain polaron pairs, which prevents the establishment of a window of net optical gain. Here we study the photophysics of poly(3-hexylthiophene) configured with different degrees of supramolecular ordering, spin-coated thin films and templated nanowires, and find marked differences in their optical properties. Transient absorption measurements evidence a partially-polarized stimulated emission band in the nanowire samples, in contrast with the photo-induced absorption band observed in spin-coated thin films. In combination with theoretical modeling, our experimental results reveal the origin of the primary photoexcitations dominating the dynamics for different supramolecular ordering, with singlet excitons in the nanostructured samples superseding the presence of polaron pairs, which are present in the disordered films. Our approach demonstrates a viable strategy to direct optical properties through structural control, and the observation of optical gain opens the possibility to the use of polythiophene nanostructures as building blocks of organic optical amplifiers and active photonic devices.

## Introduction

Conjugated polymers comprise a broad class of organic materials that have attracted a great deal of attention in the last decades due to their fundamental properties and applications in innovative optoelectronic devices^1–4^. Their flexibility, easy processability and light weight together with good charge-carrier mobilities (up to a few cm^2^V/s) enable the production of large-area, bendable or stretchable optical and electronic components via low-cost solution processing. In addition, their structural and configurational order, possibly driven by nanostructures^5,6^, critically impacts on their charge transport and optical properties^7,8^, thus providing powerful routes to finely tailor electronic processes at different scales. In this research framework, polythiophenes are frontrunners thanks to their simple synthesis and excellent charge transport. Conductive layers of poly(3-hexylthiophene) (P3HT) have been successfully integrated in a large variety of organic devices such as solar cells^9–11^, light-emitting diodes^12^, field-effect transistors^13–15^ and (bio)chemical sensors^16–18^. Recent results have demonstrated that tuning of electronic and vibronic couplings in P3HT via chemical synthesis may allow for controlling the yield of photoinduced charge formation optimizing the performance of organic-based optoelectronic devices^19^.

The electronic and optical properties of polythiophene materials and of the entire class of devices based on them are strongly influenced by the supramolecular packing of the polymer chains. Following deposition, chains can take a complex semicrystalline form which is composed of a mixture of ordered and amorphous domains^19–21^. Inside a microcrystal, P3HT chains are organized in lamellar sheets created by the interdigitation of the side chains. This causes linear arrangement of the thiophene rings along a chain and, furthermore, π-stacking of the thiophene chains in neighbouring sheets. Effective charge transport can then occur in the coherent regime, predominantly by intra-molecular band-like mechanisms along the conjugation length (intrachain)^22^. In the amorphous domains, on the other hand, the chains are folded, coiled and interlaced; the electronic structure is defined by this segmentation, the interaction between segments of neighboring chains (interchain) is now favored and charge transfer occurs randomly, in the hopping regime.

In order to control nucleation and growth of crystalline structures, different aspects, such as solvent evaporation rates^23,24^, play a fundamental role.

The regioregularity grade and the morphology can be tailored by nanofabrication approaches, such as nanostencil methods^25^, nanoimprinting^26^, electrospinning^27^, direct drawing of solvated polymers^28^ or soft lithography^23,29,30^. It is generally established that directional growth induced by confinement of polythiophene chains, resulting in higher configurational order in supramolecular packing, leads to improved charge carrier mobilities and suppression of low-frequency electronic noise^29^. However, in spite of the proven beneficial effects of highly-ordered organic nanostructures on polythiophene electronic components, little is known on their influence on the optical response^31,32^.

In this work we use steady-state and time-resolved optical spectroscopy to compare the behavior of P3HT prepared through methods leading to different nanoscale configurations, i.e. thin films (TF) realized by spin-coating, and ordered nanowires (NW) with precise spatial organization produced by solvent-assisted nanolithography. Clear evidences of the different structural order of the two configurations appear already in the steady-state absorption and photoluminescence (PL) spectra. Ultrafast transient absorption (TA) spectroscopy allows to fully disclose the inherently different character of the photoexcited carriers imparted by the different supramolecular ordering. In fact, in excited TF samples, TA spectra highlight a photo-induced absorption (PA) band due to polaron-pair states, while, surprisingly, a partially polarized stimulated emission (SE) band appears in the same energy range for NW, corresponding to optical gain from photoexcited excitons. Supported by theoretical calculations, we suggest a rationale for this difference: in ordered systems, individual polymers can be approximated as straight chains, with one-electron orbitals that extend over the whole chain. When the conjugation length is shortened as in TF, a very different scenario emerges: electrons and holes may localize on different segments of the chain favoring intra- and interchain separation of the photexcited carriers, thus explaining the significant presence of polaron pairs that is found experimentally. Our results show that the exclusive manipulation of the sample architecture can strongly influence the photophysical behavior of the resulting material, while keeping constant the chemical properties of the polymer. We define an effective strategy to tailor excited state properties of nanostructured conjugated polymers, including optical gain, in view of their application to the development of photonic devices.

## Results and Discussion

### NW fabrication and characterization

**Figure 1:**
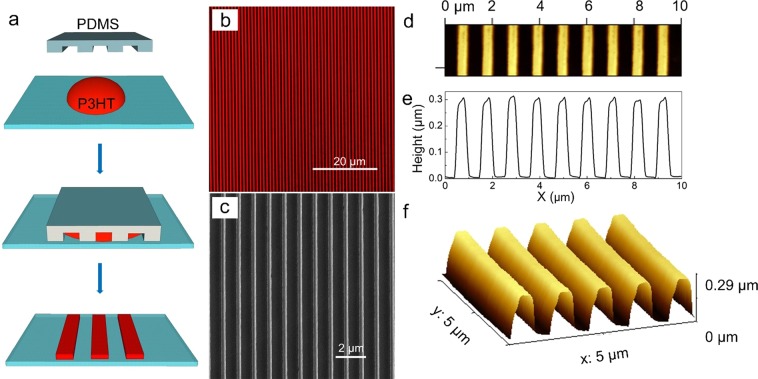
(**a**) Schematic illustration of the imprinting process used to realize P3HT NWs. Optical and morphological characterization of P3HT NWs: (**b**) confocal microscopy, (**c**) SEM and (**d**) AFM micrographs. (**e**) Height profile and (**f**) 3D AFM view of P3HT NWs.

Nanowires are realized by soft lithography through the deposition of 1 μL droplet of 0.97% wt:wt solution of regioregular P3HT in dichlorobenzene on top of a quartz substrate. An elastomeric stamp is then placed on the solution until the complete evaporation of the solvent. A scheme of the fabrication process is reported as Fig. 1a. Patterning and sample storage are entirely carried out under nitrogen atmosphere (O_2_ < 30 ppm, water vapor < 10 ppm) to avoid oxidation phenomena. After complete solvent evaporation, the stamp is removed and NWs are imaged by confocal microscopy (Fig. 1b), scanning electron microscopy (SEM) (Fig. 1c) and atomic force microscopy (AFM) (Fig. 1d–f). The pattern covers an area of about 1 cm^2^ with feature length of 5 mm. Confocal fluorescence imaging indicate the formation of bright and uniform NWs with no visible interconnecting bottom layer between adjacent P3HT wires (Fig. 1b).

Both SEM and AFM planar views confirm the formation of a high quality pattern featuring isolated NWs with linewidth of about 300 nm and period of 1 μm (Fig. 1b,c), reproducing quite faithfully the starting master. The height of the NWs is 290 ± 5 nm as captured by the height profile and 3D AFM view reported in Fig. 1e,f, respectively.

### Linear absorption and photoluminescence spectra

**Figure 2:**
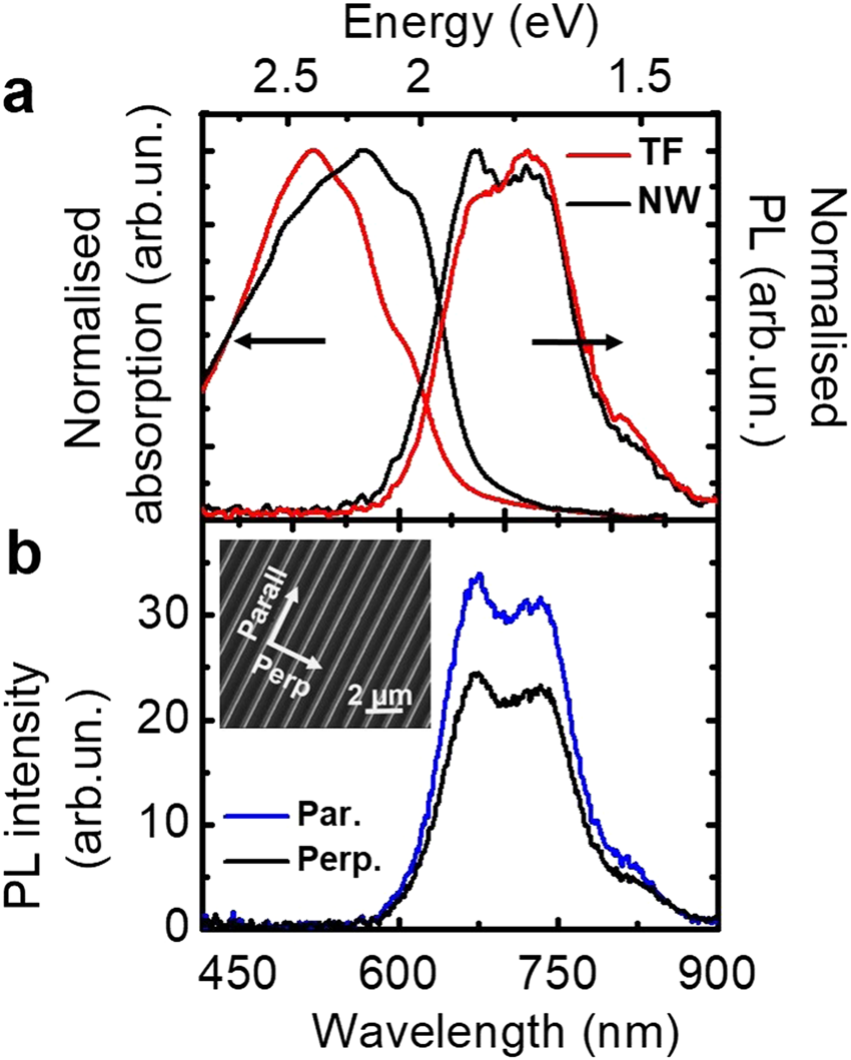
(**a**) Absorption and PL spectra on spin-cast TF and of NW made of P3HT. (**b**) PL spectra of NW excited with polarization parallel to the nanogrooves and analyzed in the two directions by polarizers placed in front of the optical fiber before PL collection.

Figure 2a shows the normalized absorption and PL spectra of both regioregular (rr) P3HT TF and NW samples. For both systems, the absorption spectra consist of a vibronic progression, with peaks at about 613 nm (2.03 eV, A_0-0_), 562 nm (2.21 eV, A_0–1_), and 520 nm (2.39 eV, A_0–2_) that reflect the transition from the lowest vibrational level of the ground state to different vibrational levels of the excited-state manifold (the subscript referring to the number of vibrational quanta ν in the initial/final states, with ν = 0, 1, 2, …), assuming that the C=C symmetric stretch at 0.18 eV dominates the coupling with the electronic states^20^. A similar progression is observed also in PL spectra, with peaks at 667 nm (1.86 eV, E_0-0_) and 736 nm (1.68 eV, E_0–1_). Notably, the main difference between the two samples lies in the relative intensity of the peaks within each progression, with both intensity ratios *I*(A_0-0_)/*I*(A_0–1_) and *I*(E_0-0_)/*I*(E_0–1_) increasing when moving from the TF to the NW sample.

These differences in the steady-state spectra of polymeric assemblies are commonly analyzed within an HJ-aggregate model^32–34^. Owing to the comparable intrachain and interchain couplings, subtle changes in the morphology of the crystalline phase of P3HT have been shown to be able to induce alternatively H-like or J-like dominant character of the spectra^33^. In line with previous results for TF spun from various solvents^20^, our absorption data for the spin-cast TF sample exhibit a dominant H-aggregate behavior, where intrachain disorder would favor the formation of molecular, Frenkel-like excitons delocalized over neighbouring chains. According to the model, the increase of the absorbance intensity ratio *I*(A_0-0_)/*I*(A_0–1_) for NW is explained by an increase of the average conjugation length with respect to the TF. For TF, we also observe a larger spectral weight in the energy region above 2.3 eV as compared to the NW, commonly attributed to absorption from amorphous domains of unaggregated polymers^20^. Furthermore, the analysis of the PL spectra within the same model suggests a J-like dominant behavior for the NW sample, where a longer conjugation length favors the formation of Wannier-Mott-like excitons delocalized over several thiophene units of a single chain^31^. In fact the intensity ratio *I*(E_0-0_)/*I*(E_0–1_) not only increases going from the TF to the NW sample, but becomes >1, which is typical of J-like aggregates. These observations are in agreement with previous observations for rrP3HT nanofibers and nanowires^31,35^. In analogy to the case of nanofibers^32^, we also measured the polarization anisotropy of the emissive species, as displayed in Fig. 2b. As a consequence of the internal ordering of the chains in the NW^23^, we find a partial anisotropy of the PL, with a dichroic ratio (*I*_*par*_/*I*_*perp*_) of ≈1.4 (where *par* and *perp* indicate respectively the PL polarization direction parallel and perpendicular to the channel), confirming that a partial alignment occurs along the channel direction due to laminar nanofluidic flow, and as a consequence, the transition moments of the molecular-conjugation segments are also mainly aligned along the fiber axis.

### Ultrafast transient spectroscopy

**Figure 3:**
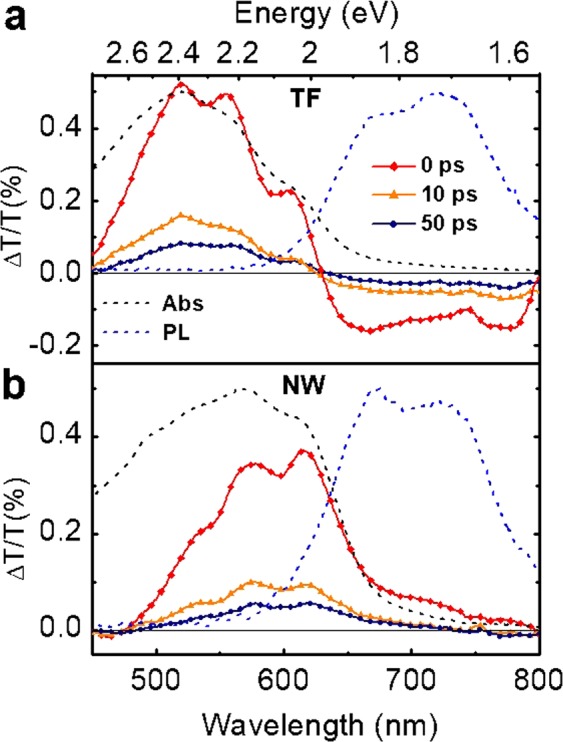
TA spectra measured on TF (**a**) and on NW (**b**), at different pump-probe delays. Dashed black and blue lines: absorption and PL spectra of the two systems, respectively. The pump and probe are with parallel polarizations. Pump fluence: 10 µJ/cm^2^.

Steady-state spectroscopies, extensively used to extract information on polymer aggregation and morphology, do not provide direct insight into the nature of primary photoexcitations and their dynamics, which are instead measured by time-resolved spectroscopies. Figure 3 shows the TA spectra of both TF (Fig. 3a) and NW (Fig. 3b) samples at different pump-probe delays, using parallel pump and probe polarizations. In the TF system, we observe a positive signal in the range between 450 and 630 nm (1.97–2.76 eV), and then a negative signal with a peak at around 670 nm (1.85 eV), i.e. below the P3HT optical gap. The TA spectra of TF of rrP3HT have been extensively investigated^19,36–40^. In agreement with previous assignment^36,39,40^, the positive band is attributed to the photobleaching (PB) of the lowest vibronic resonances of the ground state, as demonstrated by the overlap with the ground state absorption spectrum (black dashed line in Fig. 3a). The negative PA band, instead, is commonly attributed to instantaneously photogenerated polaron pairs^36,41–46^.

**Figure 4:**
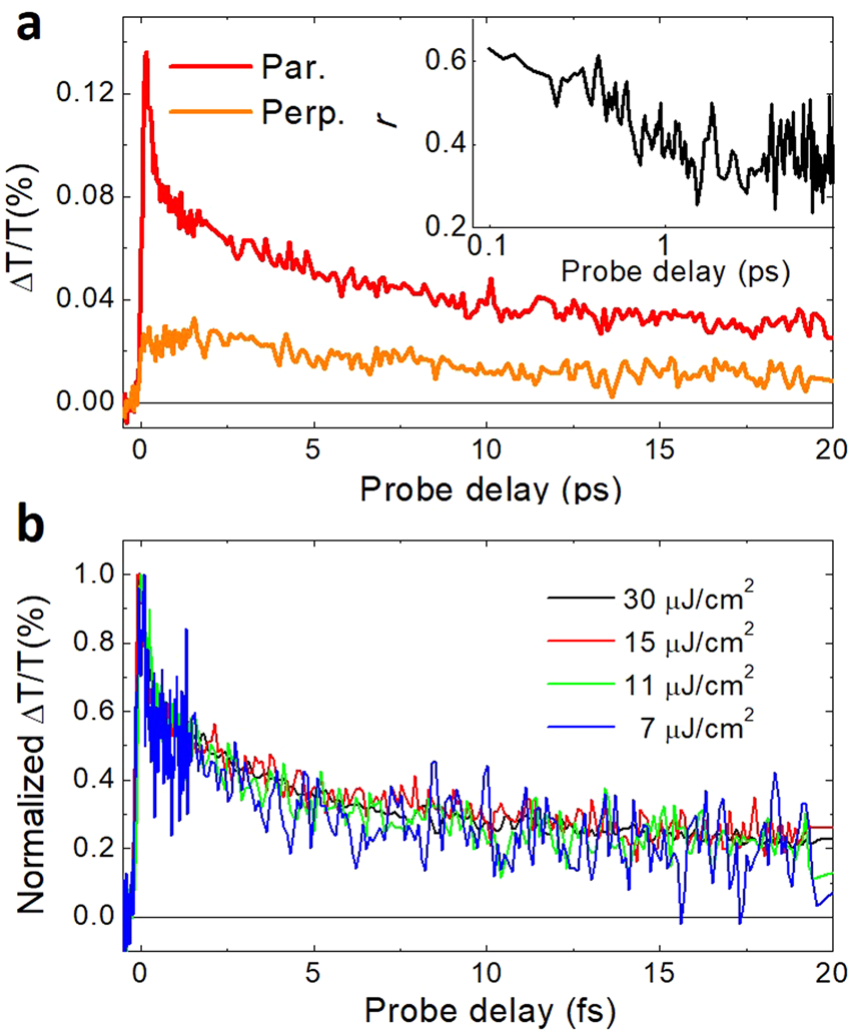
(**a**) TA dynamics at 660 nm for the NW with pump polarized parallel to their longitudinal axis, and with different probe polarizations. Pump fluence: 10 µJ/cm^2^. The inset shows the anisotropy decay. (**b**) Dynamics in the SE region (660 nm), at different pump fluences keeping the pump and probe beam with parallel polarizations.

In the NW, we see again PB in the region between 450 and 630 nm, as confirmed by the comparison of TA with the corresponding absorption spectrum (black dashed line in Fig. 3b). Looking at the below-bandgap region, however, we notice for the NW a positive signal in place of the PA negative band characterising the TF. This band can be assigned to SE from the excited state, as it matches the PL spectrum of P3HT (blue dashed line in Fig. 3b). The maximum value of gain was found to be 23.2 cm^−1^ at 670 nm, which corresponds to 100 dB/cm^47^, value comparable to other ones obtained with organic polymeric thin films^47^. To better analyse the SE signal, we studied its dependence on both polarization and pump fluence. Figure 4a compares the SE dynamics at 660 nm, obtained by using parallel and crossed polarizations between the pump and the probe beams (see also Fig. S1 in SI for TA spectra with both parallel and crossed polarisations and Fig. S2 for magic angle dynamics). We observe a higher SE signal when the probe polarization is parallel to the longitudinal axis of aligned P3HT nanowires, in agreement with the PL spectra displayed in Fig. 2b. We have also calculated the anisotropy decay (see inset Fig. 4a) by using this formula^48^:

$$r(t)=\frac{{I}_{par}\,-\,{I}_{per}}{{I}_{par}+2{I}_{per}}$$

The temporal decay of the anisotropy *r* shows an initial fast decay in around 1 ps, clearly indicating a fast depolarization of the photogenerated excitons. This confirms the fact that, even if the polymeric chains are more elongated and partially aligned, they are not isolated and an efficient interchain exciton coupling exists. TA measurements at various pump fluences are also performed with pump and probe polarizations parallel to the grooves, as shown in Fig. 4b. Our data indicate that the SE decay dynamics does not change with the pump fluence, evidencing therefore the absence of bimolecular decay processes in our excitation fluence range.

### Discussion: origin of the stimulated emission

Let us now focus on the main difference between the two samples, highlighted by the TA experiments, i.e. the appearance of SE instead of PA band in the below-gap region for the NW samples as compared to the TF case, which suggests a different nature for the primary photoexcited species in the two samples. As detailed in previous studies^36,39^, in TF samples any SE contribution is usually mostly or completely overcome by the PA signal, especially for excitation with short-wavelength/high-photon-energy pump pulses, and residual SE has been mainly attributed to singlet exciton emission from the amorphous regions^49^. A note should be done for the 0-0 SE transition (at around 670 nm) which decreases in the TF sample (H-like) not just because of the higher PA signal but also due to the decreased oscillator strength of the transition^34^.

The dynamics after photoexcitation is thus mainly dominated by photogenerated polaron pairs that can be localised over neighbouring chains in the crystalline domains. On the contrary, in NW the dominance of SE and its fast, monomolecular decay would mainly point to presence of photogenerated excitons localised on the single chain. Remarkably, up to now prominent SE bands have been only observed for regio-random (rra) P3HT^50,51^ or modified copolymers^39^, where the self-organization in ordered aggregate is less favoured. Here we observe the opposite behaviour, that is, a dominant SE signal in systems where additional long-range order is imparted by the NW morphology.

**Figure 5:**
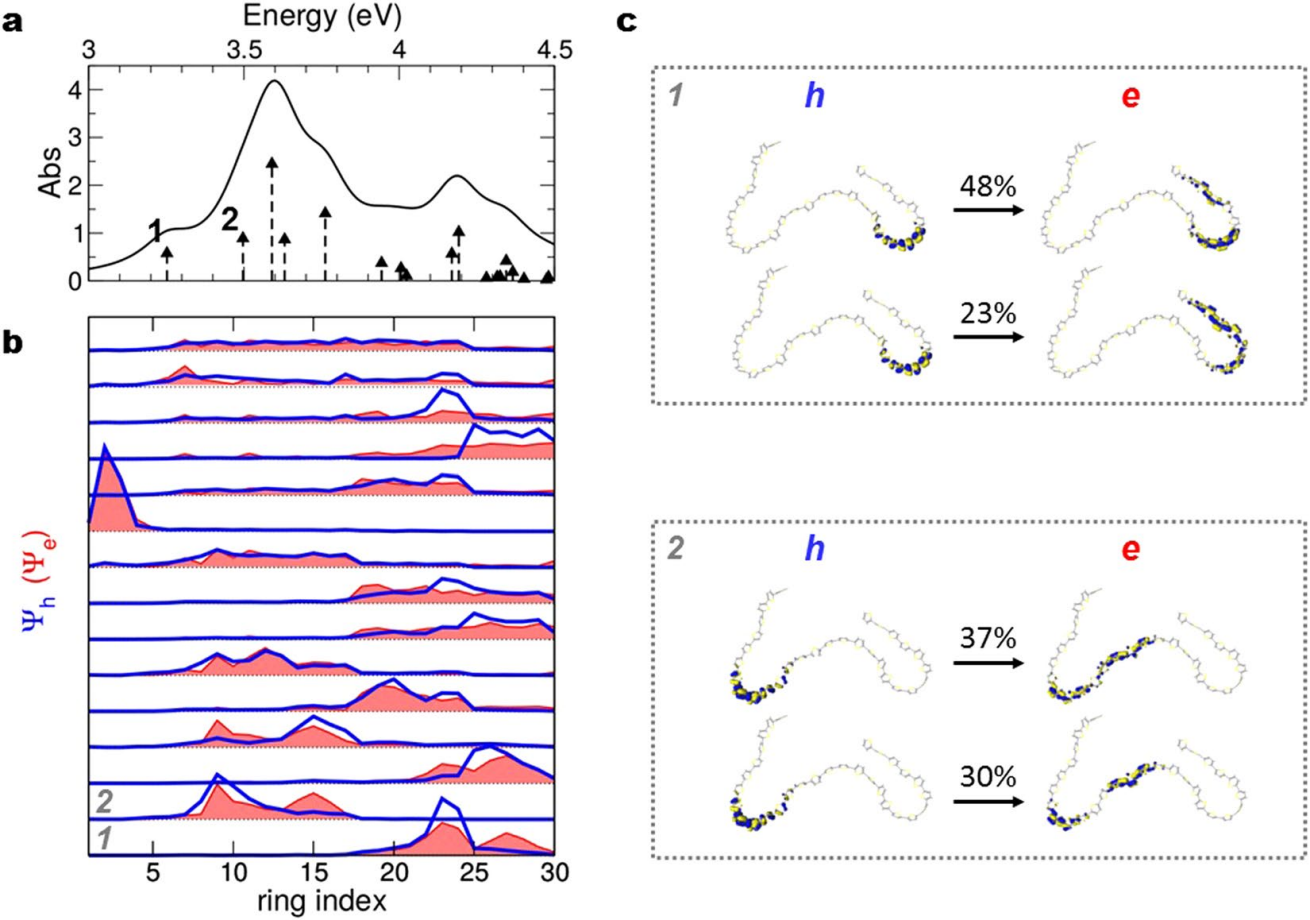
Optical properties, isolated *in vacuum*, for a thiophene chain extracted from the disordered condensate simulation. (**a**) Optical spectrum. (**b**) Ring-localization of the optical transitions above, in order of energy –occupied in blue, (hole, h), unoccupied in red, shaded area (electron, e). (**c**) For the lower-energy excited states (1 and 2), visualization of the orbitals (h, e) mostly involved in the transition (percentage of the full CI vector indicated).

To understand this apparent contradiction, we model long oligomers of 30 thiophene units, extracted from the amorphous film geometry obtained by means of classical Molecular Dynamics simulations^52^ (see Fig. S3 in SI). We consider these isolated chains, pruned to exclude side-chains that do not contribute to the relevant electronic properties, as the simplest atomistic model for the calculation of the optical properties of P3HT chains with short conjugation length. For these systems, the one-electron orbitals do not delocalize over the complete length of the chain, as they would do in straight chains with longer conjugation length, but are found to be localized on different segments of the chain (see Fig. 5c). To illustrate the effect of this particular charge distribution on the optical properties, we plot for each excitation the electron (red filled line) and hole (blue line) distribution resulting from semiempirical Configuration Interaction (CI) calculations (see Methods) on an individual chain (Fig. 5b), and the main composition of the two lowest excitations in terms of single-particle transitions (Fig. 5c). Note that these excitations, which are indicated as vertical arrows in the reference absorption spectrum (Fig. 5a), display energies that are blue-shifted with respect to experiments since calculations for the single oligomer are performed in vacuum. The lowest-energy on-chain excited states show markedly different charge distribution for the electron-hole pair, with electrons more delocalized than holes. The fact that electrons and holes are not localized on the very same rings may thus introduce separation of photoexcited carriers already at the level of the single chain.

**Figure 6:**
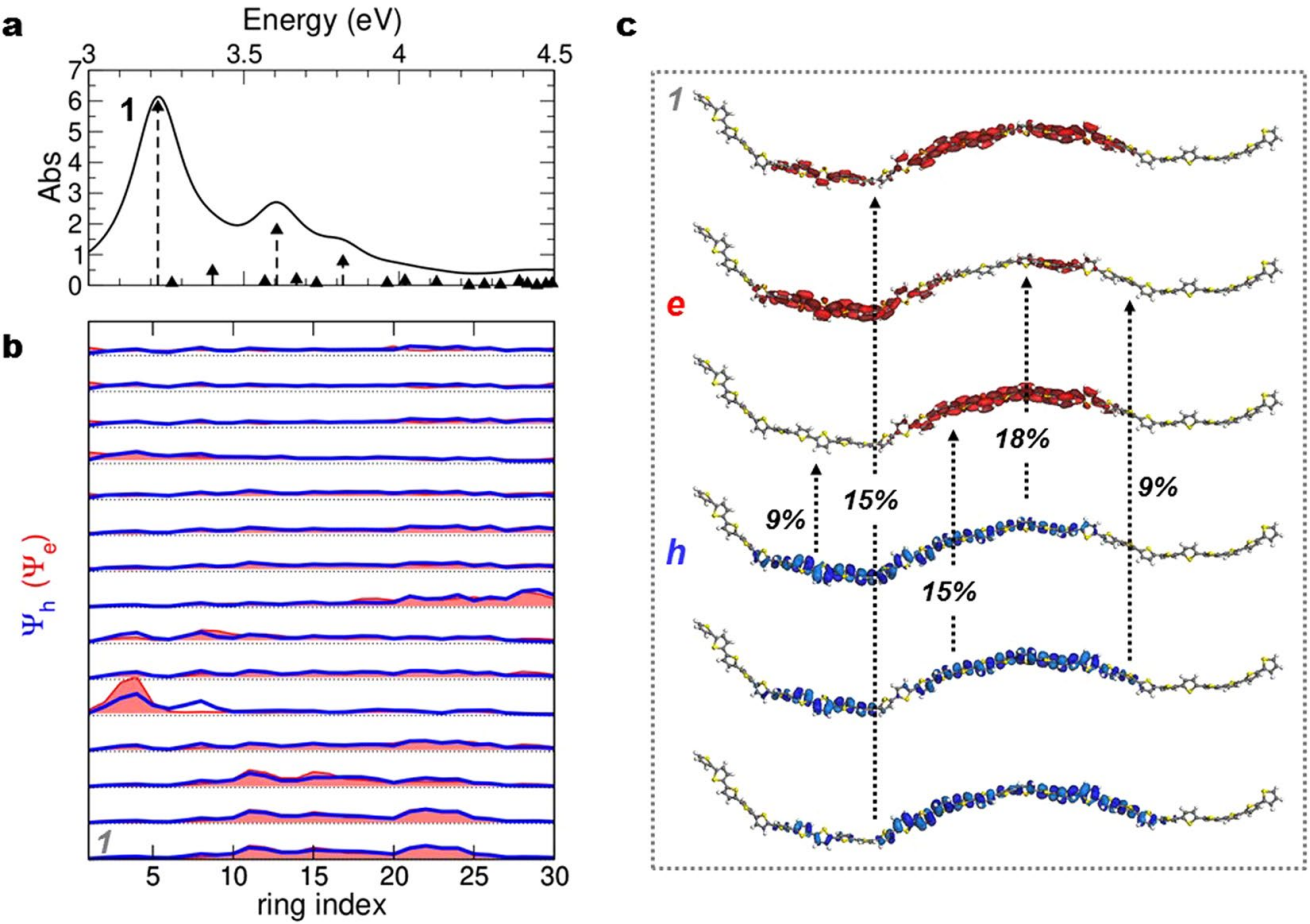
Optical properties, isolated *in vacuum*, for a thiophene chain extracted from the laminar configuration. (**a**) Optical spectrum. (**b**) Ring-localization of the optical transitions above, in order of energy –occupied in blue, (hole, h), unoccupied in red, shaded area (electron, e). (**c**) For the lowest-energy excited state (1), visualization of the orbitals (h, e) mostly involved in the transition (percentage of the full CI vector indicated).

Depending on spatial alignment of the neighbouring segments, further separation may occur as a consequence of interchain coupling (electron and hole in different chains), strongly reducing e-h recombination. A longer conjugation length, favored by the ordered arrangement of nanowires^6,23,29^, is expected to support the formation of excitons delocalized over several thiophene rings along a single chain, similarly to what predicted for crystals (~10 rings)^53^, and actually seen in our simulations for the laminar films (see Fig. 6): in this case on-chain recombination is favored. The key difference must then reside in the average conjugation length, suggesting that the reduced presence of polaron pairs in NW mostly results from suppressed intrachain localization (and inter-chain quasi-particle hopping) of photoinduced charge carriers with respect to the TF phase. The dominant transient species are thus singlet intrachain excitons superseding interchain polaron pairs and give rise to the SE signal observed experimentally for our NW samples.

### Conclusions

Ultrafast transient absorption spectroscopy discloses the nature of primary excitations governing the photophysics of P3HT under different configurational and supramolecular order. Transient absorption measurements show evidence of partially polarized stimulated emission from excitons in P3HT nanowires, which overcomes the photo-induced absorption band due to polaron pairs dominating spin-coated samples. Theoretical calculations relate this absorption to a different localization of electrons and holes over individual polymeric chains with short conjugation length, which may favor intra- and interchain separation of the carriers and subsequent photo-induced absorption from polaron pairs. Suppressing this effect by the ordered configurations of polymer chains in NW leads instead to the emergence of an optical gain band. Thus supramolecular ordering is found to critically impact not only charge transport where enhanced lateral size confinement shows a more ordered molecular arrangement and an increased field-effect mobility^23^, but also the photophysics of P3HT, and opening new perspectives for the use of polythiophene nanostructures in organic optical amplifiers and active photonic devices.

## Methods

### Solvent assisted nanolithography

TF films are prepared starting from a 0.2 mM dichlorobenzene solution of regio-regular P3HT (12.9 mg/mL, molecular weight 64.5 kDa, Aldrich), spin-cast onto quartz to obtain 100-nm thick films. High-resolution soft nanolithography for P3HT NW production is carried out by two-layer stamps comprising a thick slab (∼5 mm) of polydimethylsiloxane (PDMS) and a thin stiff layer (30–40 μm) of *h*-PDMS, an elastomer with higher Young’s modulus (∼10 MPa) suitable to define nanostructures without buckling or microcollapses^54^. Si grooved templates (masters) are realized by electron beam lithography, with 330 nm lines and 770 nm channels. *h*-PDMS is obtained by mixing 3.4 g of base (7–8% Vinylmethylsiloxane-dimethylsiloxane copolymer - ABCR) with 5 µL of modulator (2,4,6,8-tetramethyltetravinylcyclotetrasiloxane-Sigma-Aldrich) and with 18 µL of Platinum catalyst (platinum divinyltetramethyldisiloxane-ABCR). The components are mixed vigorously and degassed under vacuum. Then, 1 g of hydrosilane prepolymer (HMS-301, ABCR) is added to the mix. The pre-polymer is poured on the Si master and a thin film is achieved on the surface by spin coating (1000 rpm for 40 s). Finally, the pre-polymer is cured at 60 °C for 40 minutes. *s*-PDMS is prepared with a Sylgard 184 kit (Dow Corning), mixing the base and the curing agent in a 9:1 ratio, pouring the pre-polymer on the *h*-PDMS layer and curing at 60 °C for 1 hour.

The lithography process is performed depositing a 1 μL droplet of the same solution used for TF samples, and then placing the elastomeric stamp on the solution. Ordering and sample storage are entirely carried out under nitrogen atmosphere (O_2_ < 30 ppm, water vapor < 10 ppm) to avoid oxidation phenomena. After complete solvent evaporation, the stamp is removed and NWs are obtained on quartz (see also Fig. 1).

### Confocal, AFM and SEM micrographs

Confocal micrographs are acquired by an inverted microscope Eclipse Ti equipped with a confocal A1R-MP system (Nikon), using an Argon ion laser (excitation wavelength, *λ* = 488 nm). The sample emission is collected by a 60× (oil immersion NA = 1.40, Nikon) objectives and the fluorescence signal is detected by a spectral detection unit equipped with a multi-anode photomultiplier (Nikon).

The AFM characterization of the nanopatterned surfaces is carried out by “peak force” imaging mode in air using a Bruker Dimension Icon system equipped with a Nanoscope V controller. The used silicon tip (nominal radius of curvature of 2 nm) is mounted on silicon nitride cantilever with 0.4 N/m nominal spring constant. SEM is performed with a Nova NanoSEM 450 system (FEI), using an acceleration voltage around 8 kV and an aperture size of 30 mm.

### Absorption and polarized photoluminescence

Absorption spectra of P3HT NW and TF are collected by a double beam ultraviolet-visible spectrophotometer (Perkin Elmer). The polarized photoluminescence spectra of the NW are measured exciting the by a linearly polarized continuous wave (cw) diode laser (*λ* = 405 nm, μLS Micro Laser Systems, Inc.). The polarization of the excitation laser beam is aligned parallel to the NW longitudinal axis and the PL emission is collected by a spectrometer (USB 4000, Ocean Optics) coupled with an optical fiber with the core diameter of 200 μm. The polarization of the PL emission is characterized using a polarizer placed before the collection optical fiber.

### Ultrafast Transient Absorption Spectroscopy

The ultrafast TA setup is fed by a 100-fs, 2-kHz repetition rate Ti:sapphire system (Libra, Coherent) with a central wavelength of 800 nm. TA measurements are performed by pumping at 400 nm with the second harmonic of the laser output, generated with a 1-mm, Type I β-barium borate crystal. The pump energy is adjusted between 10 and 40 nJ, providing a fluence between 7 and 30 µJ/cm^2^. The probe pulses, with a spectrum spanning from 450 to 750 nm, are obtained by white-light generation in a 3-mm thick sapphire crystal. The measurements are performed in transmission and the probe spectrum is detected using a SP2150 Acton, Princeton Instruments spectrometer. The pump beam is modulated by a mechanical chopper at 1 kHz frequency and the differential transmission (ΔT/T) spectrum of the probe is measured as a function of probe wavelength and pump-probe delay. In order to study the polarization dependence of the sample response, the probe polarization is controlled using a half-wave plate on the beam path. The measurements on NW are performed exciting with polarization parallel to the NW longitudinal axis, while the probe polarization is changed.

### Theoretical methodology

We build models for P3HT chains in a TF by isolating single chains from an amorphous film configuration, as resulting from classical molecular dynamics (CMD)^55,56^. A view of the final structure for the simulated unit cell is illustrated in Fig. S3, while the details of CMD are reported elsewhere^52^. The cell contains 50 long chains, each of them constituted by 30 hexylthiophene units. As expected from the twisted morphology of each chain, coming from the amorphous character, the resulting electronic structure, according to our quantum semi-empirical calculations for the electronic-optical properties (see below), presents states with short conjugation lengths.

To understand the behavior of P3HT chains in NW samples, we rely both on simulations for infinite systems and crystals already present in the literature^53^, and on our ad hoc simulations performed on isolated chains of the same length as the above described ones (30 units), but obtained from a laminar film configuration^52^. The latter, having a straighter morphology, display electronic states with longer conjugation length.

Calculating electronic and optical properties for the full cell is not feasible, so we adopt a simple model that allows us to gain information on this complex configuration. From the morphology in the condensed cell we extract the structural conformation of each isolated chain, Fig. S3a. Since it is known (and also found here for shorter “cuts” of the full chains) that the hexyl side-chains introduce localized electronic states far from the frontier energies, the chain is then cleaned and H-saturated at the C_3_ site, as also shown in Fig. S3b,c for one P_30_T. To obtain the orbital distribution and optical spectra of each chain, we use semi-empirical Hartree-Fock plus many-body CI calculations including single excitations only, according to the ZINDO/S model^57^. This approach is known to be reliable for simulating the optical properties of these polymeric materials^58^.

## Supplementary information

Revised Portone SI
