# Supplementary material for: Tailoring optical properties and stimulated emission in nanostructured polythiophene
Source: Sci Rep. 2019 May 14;9:7370. doi: 10.1038/s41598-019-43719-0 (PMC6517583; doi:10.1038/s41598-019-43719-0)
Supplement: Supplementary file 1 — Revised Portone SI [file 41598_2019_43719_MOESM1_ESM.docx]

**Supplementary Information**

**Tailoring optical properties and stimulated emission in nanostructured polythiophene**

Alberto Portone^1,2,**^, Lucia Ganzer^3,**^, Federico Branchi^3, ‡^, Rodrigo Ramos^4,†^, Marília J. Caldas^4^, Dario Pisignano^2,5^, Elisa Molinari^6,7^, Giulio Cerullo^3^, Luana Persano^2*^, Deborah Prezzi^6*^ and Tersilla Virgili^8*^

^1^ Dipartimento di Matematica e Fisica “Ennio De Giorgi”, Università del Salento, Via Arnesano I-73100, Lecce, Italy

^2^ NEST, Istituto Nanoscienze-CNR and Scuola Normale Superiore, Piazza S. Silvestro 12, I-56127 Pisa, Italy. Email: [luana.persano@nano.cnr.it](mailto:luana.persano@nano.cnr.it)

^3^ Dipartimento di Fisica Politecnico di Milano, I-20132 Milano, Italy

^4^ Instituto de Física, Universidade de São Paulo, 05508-900 São Paulo, SP, Brazil

^5^ Dipartimento di Fisica, Università di Pisa, Largo B. Pontecorvo 3, I-56127 Pisa, Italy

^6^ Istituto Nanoscienze CNR-NANO-S3, Via Campi 213/A, I-41125 Modena, Italy. Email: [deborah.prezzi@nano.cnr.it](mailto:deborah.prezzi@nano.cnr.it)

^7^ Dipartimento di Scienze Fisiche, Informatiche e Matematiche, Università di Modena e Reggio Emilia, Via Campi, 213/a, I-41125 Modena, Italy

^8^ IFN-CNR, c\o Dipartimento di Fisica, di Milano, I-20132 Milano, Italy. Email: [tersilla.virgili@polimi.it](mailto:tersilla.virgili@polimi.it)

^**^ These authors equally contributed to the work

^†^Current address: Centro Universitario das Faculdades Metropolitanas Unidas, São Paulo, SP, Brazil

^‡^Current address: Max Born Institute, Max-Born-str. 2A,

12489 Berlin, Germany

***Ultrafast transient absorption spectroscopy***

TA measurements were acquired with different combinations of pump and probe polarisations, obtained by placing two half-wave plates on the beams paths. TA spectra at different pump-probe delays (with pump polarization aligned to the longitudinal axis of P3HT NW) are shown in Figure S1: NW samples in the upper panels (a,b) and TF in the lower ones (c,d).

**Figure S1.** TA spectra of NW (a,b) and TF (c,d) samples at different probe delays, in the visible range. (a), (c) measurements are acquired with pump and probe polarisations parallel, while (b), (d) with cross polarisations between the two beams. Pump fluence 10 µJ/cm^2^.


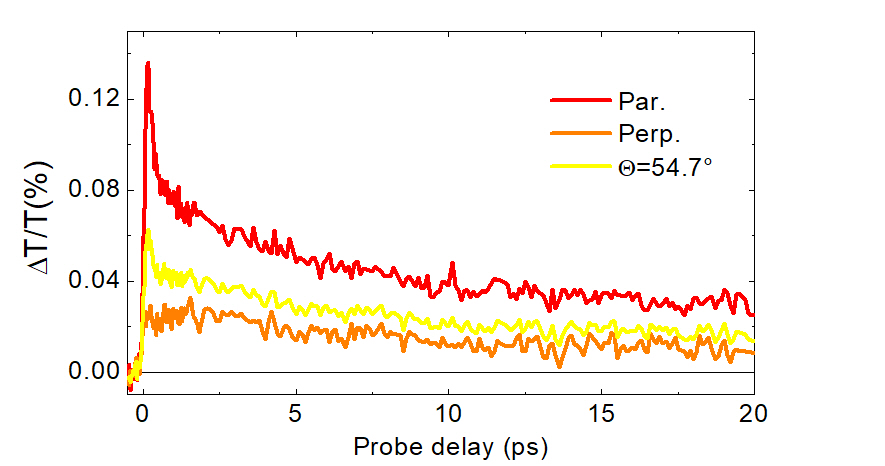


**Figure S2.** TA dynamics at 660 nm for the NW with different polarizations between pump and probe; magic angle dynamics has been calculated from experimental parallel and perpendicular dynamics. Pump fluence: 19 µJ/cm^2^.

***Theoretical methodology***


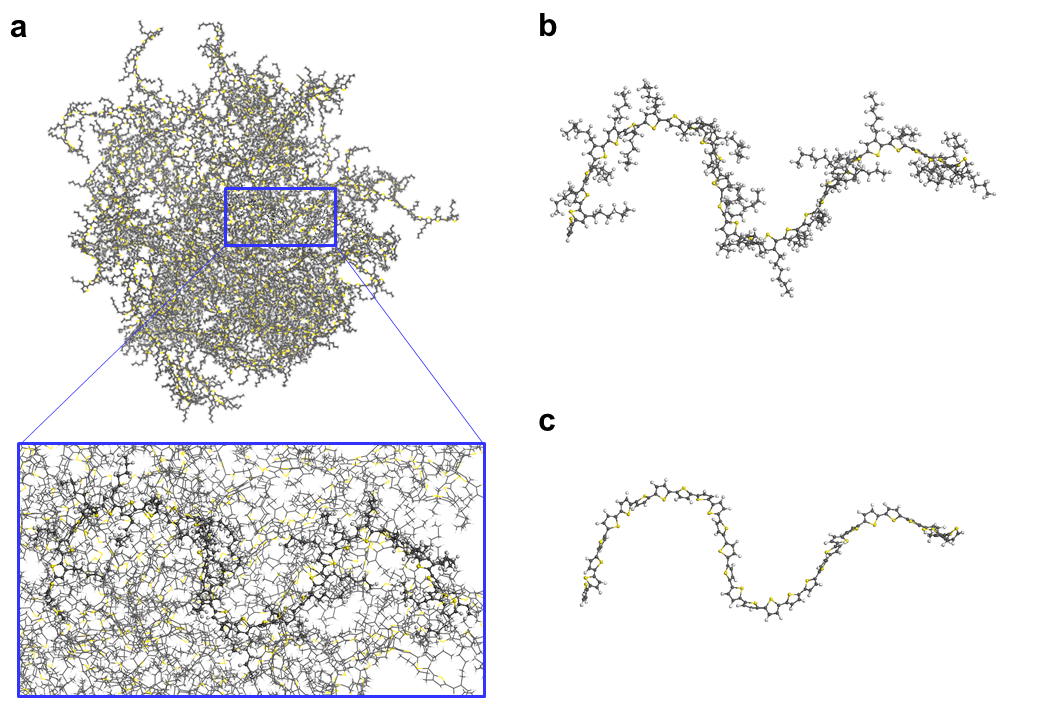


**Figure S3.** Visualization of the procedure to extract from a P3HT condensate the geometric structure of a clean poly-thiophene chain, used for the electronic structure calculations: (a) full amorphous cell highlighting (black) one specific chain; (b) hexyl-thiophene chain, as extracted from the full cell simulation; (c) thiophene chain, after optimized H-saturation of the carbon atoms previously linked to the removed hexyl sidechains.
